# Nanopore sequencing for smear-negative pulmonary tuberculosis—a multicentre prospective study in China

**DOI:** 10.1186/s12941-024-00714-2

**Published:** 2024-06-14

**Authors:** Xiaojing Yan, Guoli Yang, Yunfei Wang, Yuqing Wang, Jie Cheng, Peisong Xu, Xiaoli Qiu, Lei Su, Lina Liu, Ruixue Geng, Yingxia You, Hui Liu, Naihui Chu, Li Ma, Wenjuan Nie

**Affiliations:** 1 Medical Quality Control Center, Beijing Chest Hospital, Capital Medical University, Beijing, 101149 PR China; 2 Tuberculosis Department, Tuberculosis Hospital of Jilin Province (Jilin Provincial Infectious Disease Hospital), Changchun, 130500 PR China; 3 Department of Medicine, Hangzhou Shengting Medical Technolog, Ltd, Zhejiang Hangzhou, 310000 PR China; 4 The Fourth People’s Hospital of Qinghai Province, Xining, 510650 PR China; 5 Tuberculosis Department, Anhui Provincial Chest Hospital, Hefei, 230022 PR China; 6 Tuberculosis Department, Henan Province Anyang City Tuberculosis Prevention and Control Institute, Henan Province, Anyang City, 455000 PR China; 7 Tuberculosis Department, Hengshui Third People’s Hospital, Hengshui City, Henan Province 053099 PR China; 8 Tuberculosis Department, Hohhot Second Hospital, Hohhot City, Inner Mongolia Autonomous Region 010020 PR China; 9 Tuberculosis Department, Zhengzhou Sixth People’s Hospital, Zhengzhou City, Henan Province 450015 PR China; 10 Tuberculosis Department, Beijing Chest Hospital, Capital Medical University, Beijing, 101149 PR China; 11 Department of medical oncology, Beijing Chest Hospital, Capital Medical University, Beijing, 101149 PR China

**Keywords:** Nanopore sequencing assay, Pulmonary tuberculosis, Accuracy, Molecular diagnostic techniques

## Abstract

**Purpose:**

In this prospective study, the diagnosis accuracy of nanopore sequencing-based *Mycobacterium tuberculosis* (MTB) detection was determined through examining bronchoalveolar lavage fluid (BALF) samples from pulmonary tuberculosis (PTB) -suspected patients. Compared the diagnostic performance of nanopore sequencing, mycobacterial growth indicator tube (MGIT) culture and Xpert MTB/rifampin resistance (MTB/RIF) assays.

**Methods:**

Specimens collected from suspected PTB cases across China from September 2021 to April 2022 were tested then assay diagnostic accuracy rates were compared.

**Results:**

Among the 111 suspected PTB cases that were ultimately diagnosed as PTB, the diagnostic rate of nanopore sequencing was statistically significant different from other assays (*P* < 0.05). Fleiss’ kappa values of 0.219 and 0.303 indicated fair consistency levels between MTB detection results obtained using nanopore sequencing versus other assays, respectively. Respective PTB diagnostic sensitivity rates of MGIT culture, Xpert MTB/RIF and nanopore sequencing of 36.11%, 40.28% and 83.33% indicated superior sensitivity of nanopore sequencing. Analysis of area under the curve (AUC), Youden’s index and accuracy values and the negative predictive value (NPV) indicated superior MTB detection performance for nanopore sequencing (with Xpert MTB/RIF ranking second), while the PTB diagnostic accuracy rate of nanopore sequencing exceeded corresponding rates of the other methods.

**Conclusions:**

In comparison with MGIT culture and Xpert MTB/RIF assays, BALF’s nanopore sequencing provided superior MTB detection sensitivity and thus is suitable for testing of sputum-scarce suspected PTB cases. However, negative results obtained using these assays should be confirmed based on additional evidence before ruling out a PTB diagnosis.

## Introduction

The World Health Organization (WHO) reported that there are about 10 million people are living with tuberculosis (TB) global [1]. In estimated TB cases, globally, cases of pulmonary TB (PTB) only account for 8.5%, but, in China, PTB is one of the most prevalent forms of TB. To delay the development of PTB and reduce PTB-related high morbidity and mortality, its early diagnosis and management are essential.

Mycobacterial growth indicator tube (MGIT) culture-based and Xpert-based Mycobacterium tuberculosis (MTB)-detection assays are the most important clinical TB diagnostic assays used during the last decade. In fact, MGIT culture remains the most commonly used diagnostic methodology for PTB, although the sensitivity of this assay is at most 50% when it is used to test bronchoalveolar lavage fluid (BALF) or sputum samples. Moreover, MGIT culture testing requires a long incubation period that has driven the development of molecular assays that provide rapid results in a short time (within hours). Currently used rapid molecular MTB-detection assays include Xpert MTB/RIF (Cepheid) and loop-mediated isothermal amplification (LAMP) assays, the latter of which is based on the transcription-reverse transcription concerted reaction (TRC). Pooled results obtained from 25 studies reveal that the overall sensitivities of 93% and 89% and specificity rates of 94% and 98% for LAMP and Xpert MTB/RIF, respectively. These results together with their rapid turn-around times and potential use as high-throughput, automated assays have prompted researchers to recommend their use as initial PTB diagnostic assays.

New detection technologies, like nanopore sequencing, have markedly reduced the cost and run times of detection [2]. Nanopore sequencing utilises highly efficient, accurate and effective high-throughput sample processing that through the analysis of DNA fragments (thousands to billions) which single and simultaneous sequenced to enable rapid testing of microorganisms. However, to date this method has only been used to detect MTB in sputum samples, despite the fact that many suspected TB patients cannot produce sputum. To address this issue, here mycobacterial pathogens were detected in BALF samples collected from PTB-suspected patients based on sequencing of polymerase chain reaction (PCR) products with specific DNA sequences for the M. tuberculosis complex, thus to confirm nanopore sequencing’s diagnosis reliability. These results were then in comparison with the corresponding results obtained using MGIT culture and Xpert MTB/RIF assays.

## Materials and methods

### The enrollment of patients

This study was approved by the Ethics Committee of Beijing Chest Hospital. All methods were carried out in accordance with guidelines and regulations of Beijing Chest Hospital. Informed consent was obtained from all subjects or their legal guardian. Specimens for this multicentre clinical study were obtained from suspected PTB cases from four different provincial regions of China, namely Beijing, Anhui, Qinghai and Jilin. Sociodemographic information and clinical data were collected from enrolled patients from September 2021 to April 2022. Thereafter, PTB was diagnosed based on clinical findings and MTB-detection results in the patients’ BALF samples. Included study cases need to meet one of the criteria below: (1) presentation of TB symptoms, such as fever, subacute cough, night sweats and/or loss of weight; (2) patchy shadows or miliary pulmonary nodules were observed on chest X-ray. Clinicians diagnosed PTB cases in accordance with the clinical guidelines approved by the Ministry of Health of the People’s Republic of China; (3)all specimen smears were negative for acid-fast bacterial stains; (4) new suspected smear-negative TB cases.

### Clinical specimens

155 BALF specimens were collected between September 2021 to April 2022. The samples were kept at − 70 °C until DNA extraction was required. All specimens were tested negative using Ziehl-Neelsen staining.

### DNA extraction

Genomic DNA (gDNA) from 10 ml BALF was isolated using centrifugation, which was a previously reported established method [3, 4]. Take the following steps to deal with it: (1) Pre-treatment steps: Take 10 ml of the sample from a 15 ml centrifuge tube and centrifuge at 4000 rpm for 5 min. Discard the supernatant (retain 500ul) and rinse the precipitate with the remaining 500ul in the tube to obtain the liquid. Add lysozyme and wall lysozyme, mix well, and treat at 30 ° C for 15 min. After finishing, add protease K and 0.05 mm zirconia grinding beads, and use a grinder for grinding. (2) Nucleic acid extraction: Use QIAampDNAMicrobiomeKit (QIAGE, Canada) to extract nucleic acid from the sample according to the instructions; Use QubitdsDNA high-sensitivity detection kit to determine DNA concentration. Next, the purity of the preparation was then determined utilizing a Nanodrop spectrophotometer (Thermo Fisher Scientific). Samples with OD260/280 values around 1.8 and values of OD260/230 within 2.0-2.2 can be used for subsequent experiments.

### Polymerase chain reaction (PCR)

To detect MTB, DNA sequences of four MTB genes, (i.e., IS6110, rpoB, hsp65 and gyrB ) were subjected to targeted amplification. All primers were mixed to generate a primer pool to simultaneously amplify all target genes.The primers were rpoB-For: TGTTGGACATCTACCGCAAG, rpoB-Re: CGAGACGTCCATGTAGTCCA; hsp65-For: TCGAGACCAAGGAGCAGATT, hsp65-Re: GCGAGCAGATCCTCGTAGAC; gyrB-For: CGAAACCACGGAATACGACT, and gyrB-Re: GTTGTGCCAAAAACACATGC. And we used multiple PCR to simultaneously amplify all target genes.The rifampicin (RIF) resistance-determining region (RRDR) sequence of rpoB mainly involved in rifampicin resistance and thus was amplified here in order to identify RIF-resistant MTB to guide patient treatment. For testing of 155 collected samples, PCR mixtures were first prepared according to the protocol of the LongAmp Taq 2x Master Mix Kit (#M0287, NEB, USA). In brief, sample gDNA (20 ng), forward and reverse primer [5] were IS6110-For: CTGAACCGGATCGATGTGTA, and IS6110-Re GGTGGTTCATCGAGGAGGTA, and were stock solution (10 µM, 5 µL of each) and LongAmp Taq 2× Master Mix (15 µL) were prepared into a 30 µL PCR reaction mixture. PCR reactions were: (1) 94 °C for 2 min, (2) 30 s at 94 °C, 45 s at 60 °C for and 1 min at 72 °C as one cycle total cycling for 30 times and (3) finally held at 4 °C. Adjust the PCR product concentration to a final concentration ranging from approximately 100 to 200 fmol per µl by gradient dilution and mix together equimolar amounts of PCR products. In order to confirm the reliability of the nanopore sequencing results, we also used hsp65 and gyrB genes as the basis for distinguishing the Mycobacterium tuberculosis complex and NTM. The gene of rpoB was used for detection of drug resistance.

### Library preparation and sequencing of nanopore

Ligation Sequencing Kit (SQK-LSK109; Oxford Nanopore Technologies (ONT), Oxford, UK) and Native Barcoding Kit (EXPNBD104 and EXP-NBD114; ONT) were utilized to ligate multiplex PCR amplicons derived from 155 samples each to barcoding sequences. Follow the Native Barcoding Kit instructions to dilute sample (100PN to 200 fmol) in nuclease-free water (65 µL) while performing an around 3 h-reaction of amplicon end preparation and native barcode ligation. Subsequently, for generation of containing 50 to 100 fmol of DNA’s final adapter-ligated DNA library, the ligation kit (NEB) and Agencourt AMPure XP beads (Beckman Coulter, USA) were applied to perform the adapter ligation and cleaning steps, respectively. Next, load the library into an R9.4 flow cell (ONT) with enough validated pores (≥ 800 pores) and followed by DNA sequencing with a GridION instrument (ONT). Next, clean the flow cell in accordance with the manufacturer’s instructions for the Flow Cell Wash Kit (EXP-WSH004; ONT) after the sequencing reaction is done, and then keep at 4 °C until use.

### Data analysis of nanopore

Guppy software (version 4.5.2, ONT) [6] was applied to analyse the raw nanopore data (fast5). The “--config dna_r9.4.1_450 bps_hac.cfg–num_callers 4 --cpu_threads_per_caller 4” parameter was used for repeating the basecalling for data, followed by applying “--barcode_kits EXPNBD104 EXP-NBD114” parameter for barcode recognition and the “--config configuration.cfg–trim_barcodes” parameter was utilized for trimming sequences. Then, NanoPlot (version 1.28.1) [7] and medaka (version 1.3.2) [8] were applied for counting the sequence data and recognizing variant calls, respectively.

Lastly, mapping the raw reads to the MTB H37Rv genome reference sequence, and Genomics software was then applied for assembling the trimmed reads onto the reference genome.

The quality control of nanopore sequencing results mainly involves the following steps. Firstly, The raw data was converted into Fastq file with the base calling software Guppy. Then, repeated sequences and low-quality sequencing data (including quality score Q < 9 and sequencing length < 200nt or > 2000nt) were removed. Finally, Sanger sequencing was utilized to confirm that the mutation frequency detected by nanopore sequencing was lower than 50% of SNP or lower than 80% of INDEL.

### Consensus sequence-based sequencing variant identification for use in evaluating nanopore sequencing accuracy

On the basis of Sanger sequencing, the sequences of the assembled nanopore were compared to the reference sequence Sanger by ClustalW thus to determine the nanopore sequencing’s detection reliability. To ensure the reliability of the nanopore sequencing method, the percentage identity of each alignment was identified [8–10].

### Statistical analysis

Microsoft Excel and SPSS software were respectively applied for data entry and statistical analysis. Comparison of diagnostic accuracy differences between the nanopore sequencing assay were performed by McNemar’s and the other two methods, while Fleiss’ kappa values were determined to compare diagnostic accuracy consistency rates between nanopore sequencing, MGIT culture and Xpert MTB/RIF. Plotting receiver operating characteristic curves to compare the performance of three methods. By calculating Youden’s index, area under the curve (AUC) values, sensitivity, specificity, accuracy, positive predictive value (PPV) and negative predictive value (NPV) to evaluate PTB diagnostic performances of the three assays.

## Results

### Participants

**Fig. 1:**
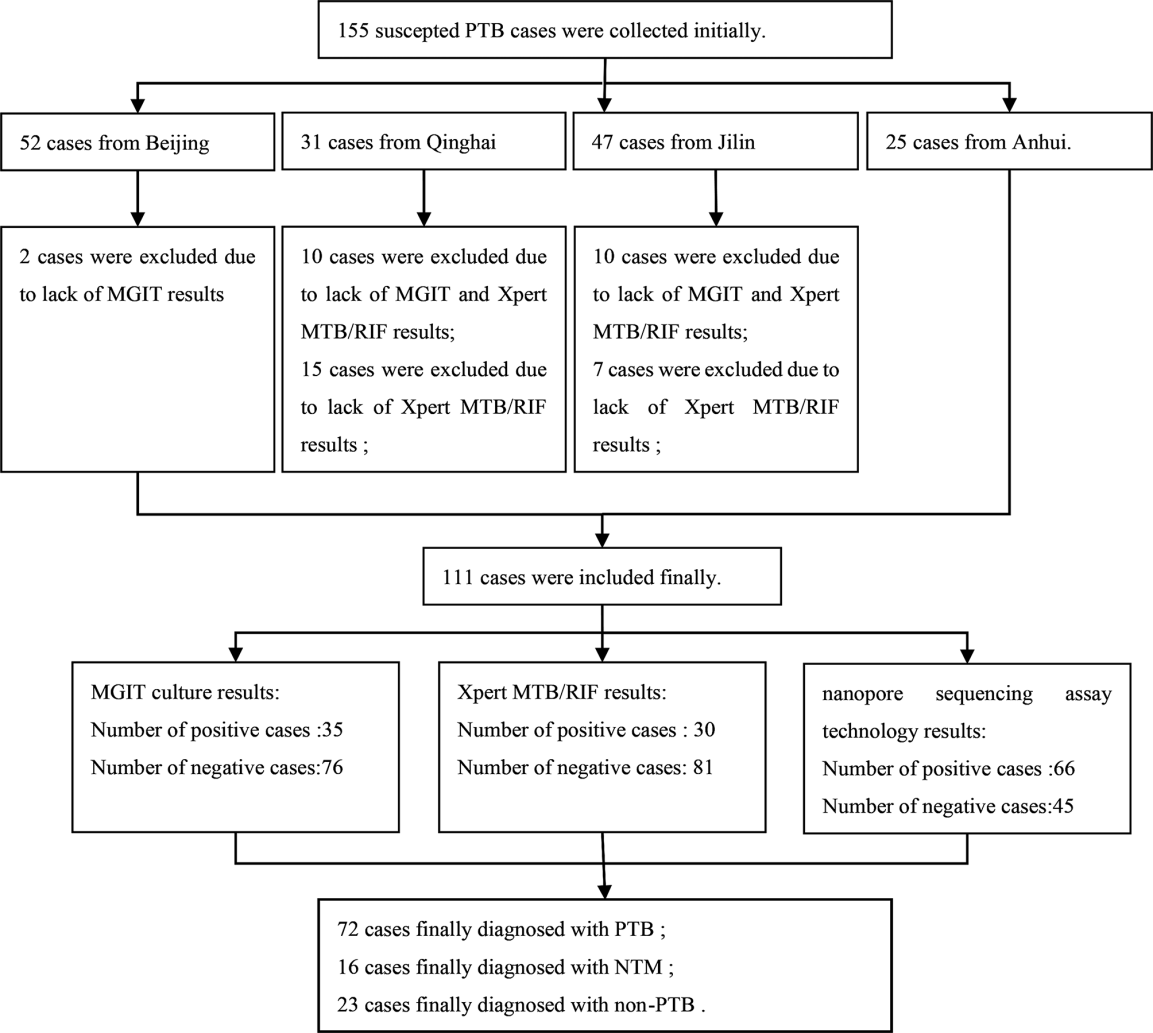
Patients collected in this study

Initially, there were 155 PTB-suspected cases were enrolled in this study, but 44 of them were excluded because of missing MGIT culture or Xpert MTB/RIF results. Confirmed diagnosis based on clinical assessment combined with radiological findings and diagnostic results using a composite reference standard (CRS).Definite TB: Clinicians diagnosed TB cases according to approved clinical guidelines of the Ministry of Health of the People’s Republic of China. Diagnosed PTB cases complied with one or more of the following criteria: (1) positive microbiological results (including acid-fast smear staining results or culture of Mycobacteria tuberculosis from specimens); (2) pathological lung tissue biopsy results consistent with pathological features of TB; (3) reduced lesion size or disappearance of lesion(s) after 3 months of anti-TB treatment. Otherwise, cases were classifed as non-TB cases. Sociodemographic characteristics, clinical diagnostic and laboratory testing information are presented in Table 1. A total of 72 male patients completed the study, accounting for 64.9% of patients. Patients’ average age was 49.8 years (age range 1-100 years). The process used to diagnose patients is outlined in Fig. 1, with final PTB diagnoses obtained for 72 patients, NTB diagnoses obtained for 16 patients and non-PTB diagnoses obtained for 23 patients, as listed in Table 1.

**Table 1:** Characteristics of patients enrolled in the study

Characteristic	No.of patients(%)(*N* = 82)
Age, years, mean(range)	49.8(1-100)
Male sex, n(%)	72(64.9)
Disease diagnosis	
PTB	72(64.9)
NTM	16(14.4)
non-PTB	23(20.7)
nanopore sequencing assay technology	
positive	66(59.5)
negative	45(40.5)
MGIT culture	
positive	35(31.5)
negative	76(68.5)
Xpert MTB/RIF	
positive	30(27.0)
negative	81(73.0)

### Nanopore sequencing performance in comparison with MGIT culture or Xpert MTB/RIF

We compared performance rates obtained from the nanopore sequencing assay with the other two assays, as determined based on McNemar’s test results and Fleiss’ kappa values. Regarding the McNemar’s test, a P value of < 0.05 for the performance result difference was regarded as statistically significant. With regard to the Fleiss’ kappa value, values within specific ranges were interpreted as follows: 0 ~ 0.20 as slight consistency, 0.21 ~ 0.40 for fair consistency, 0.41 ~ 0.60 as moderate consistency, 0.61 ~ 0.80 for substantial consistency and 0.81 ~ 1 as almost perfect consistency. Results including positive and negative MTB detection-based performance rates and Fleiss kappa values for nanopore sequencing versus the MGIT culture assay are shown in Table 2 and versus Xpert MTB/RIF assay was presented in Table 3. These results revealed statistically significant performance rate differences between the nanopore sequencing assay and the other two assays (*P* < 0.05), as well as Fleiss’ kappa consistency values between nanopore sequencing and MGIT culture assays of 0.219 (Table 2), indicating fair results consistency, and between nanopore sequencing and Xpert MTB/RIF assays of 0.303 (Table 3), indicating fair results consistency.

**Table 2:** Performance of nanopore sequencing assay technology and MGIT culture

**Nanopore sequencing assay technology**	MGIT culture		χ2	*P* value	Fleiss’ kappa value
	Positive cases	Negative cases			
positive cases	29	37	1.000	0.000	0.219
negative cases	6	39			

**Table 3:** Performance of nanopore sequencing assay technology and Xpert MTB/RIF

**Nanopore sequencing assay technology**	Xpert MTB/RIF		χ2	P value	Fleiss’ kappa value
	Positive cases	Negative cases			
positive cases	29	37	6.000	0.000	0.303
negative cases	1	44			

**Fig. 2:**
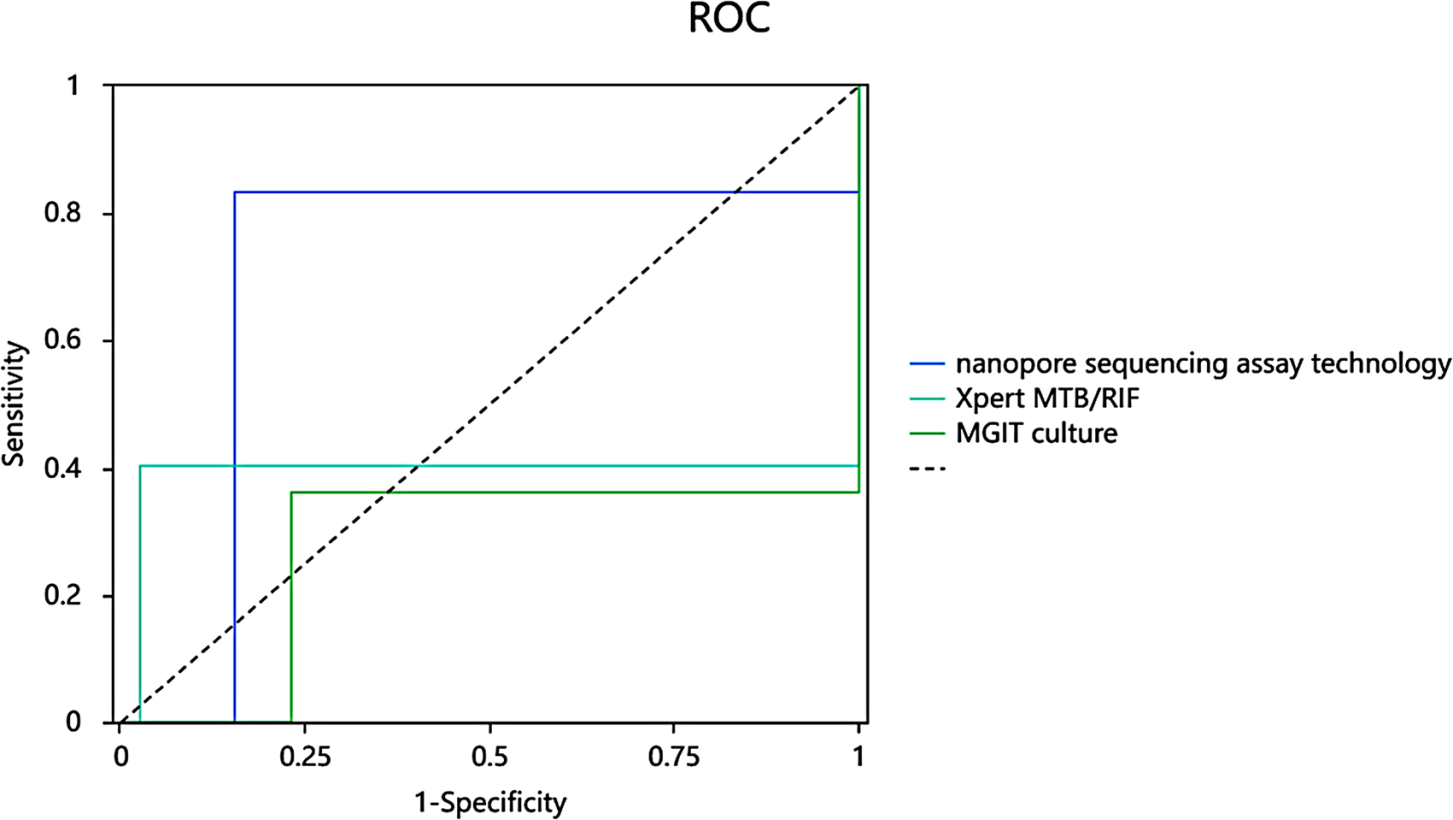
Receiver operating characteristic curves of nanopore sequencing assay technology, Xpert MTB/RIF and MGIT culture

The results obtained in this study also revealed that for MTB detection, nanopore sequencing was more sensitive (83.33%) than MGIT culture (36.11%) and Xpert MTB/RIF (40.28%) (Table 4), while Xpert MTB/RIF provided greatest specificity. Results obtained for Youden’s index, accuracy and NPV indicated superior performance of nanopore sequencing performance, with Xpert MTB/RIF performance ranking second. The AUC value of nanopore sequencing (0.840, 95% CI: 0.757, 0.922) were greater than those of exceeded Xpert MTB/RIF (0.689, 95% CI: 0.592,0.785) and MGIT culture (0.565, 95% CI: 0.455, 0.675) assays (Table 4). Above results demonstrated that the nanopore sequencing assay could offer greater MTB detection accuracy than corresponding accuracies obtained using the other two assays for the diagnosis of suspected PTB cases. AUC curves are shown in Fig. 2.

**Table 4:** Performance indicators of test accuracy evaluation

Methods	Sensitivity	Specificity	Youden’s index	Accuracy	PPV	NPV	AUC
MGIT culture	36.11%	76.92%	0.13	50.45%	74.29%	39.47%	0.565
Xpert MTB/RIF	40.28%	97.44%	0.38	60.36%	96.67%	46.91%	0.689
nanopore sequencing assay technology	83.33%	84.62%	0.68	83.78%	90.91%	73.33%	0.840

## Discussion

Early stage active TB disease is often missed by diagnosticians owing the absence of symptoms of TB. To address this issue, radiological and immunological testing of sputum or BALF may be conducted for detecting TB cases. Nevertheless, clinicians are usually unwilling to initiate early empiric anti-TB therapy simply on the basis of radiological and immunological test results when patients are unable to produce sputum. Thus, there is an urgently needed to develop new diagnostic techniques to help clinicians diagnose sputum-scarce TB patients correctly.

As compared with previous studies demonstrating good pathogen detection results of clinical sputum samples based on nanopore assay, here we assessed the diagnostic value of BALF samples using nanopore sequencing, a method that to date has mainly been applied to genomic DNA sequencing [11]. With advances in sequencing chemistry and computational power, in recent years, nanopore sequencing assay has been increasingly used for clinical applications [12]. For example, such assays have been extensively used to diagnose patients with fever who lack TB culture-positive results and detectable localised infections [13, 14] and have enabled rapid diagnosing of patients with slow-growing microorganisms-caused infections by such as TB and non-tuberculous mycobacteria (NTM) [15–20].

It is of great importance to rapidly identify mycobacteria at the species level to differentiate between TB and NTM infections in order to select appropriate medications for patient treatment that can minimise unnecessary testing and unnecessary drugs-related side effects, reduce overall cost of therapy and maximise treatment outcomes. For this reason, sequence-based assays requiring at most a few days to complete, and it greatly affects the clinical diagnosis of TB, as clinical management of mycobacterial infections that has traditionally depended on sample testing by private laboratories [2]. In fact, nowadays a single sequencing run can process more than 10 samples, dramatically reducing the cost of sequencing individual samples, which was prohibitively high at the time nanopore sequencing assays were initially utilized to diagnose infectious diseases [2]. Indeed, the MinION sequencing device developed by ONT has become increasingly popular due to its advantages, such as low device cost, short run time and small size that easy portability [7, 8] that have enabled increasing numbers of clinical laboratories to adopt this platform for use in conducting diagnostic nanopore sequencing assays. In 2015, the device was first made commercialized, it had a high sequencing error [21]. However, after the device underwent several rounds of re-engineering, its sequencing error rate decreased to an acceptable level thus the market demand for the device surged [22, 23]. In turn, this increase in demand has boosted efforts to further improve device scalability, convenience and flexibility that have enabled the improved MinION devices to serve clinical microbiology laboratories better and dramatically transform clinical microbiological testing [2].

The detection of fungi, bacteria, parasitic organisms and viruses has been facilitated due to the next-generation sequencing through untargeted DNA/RNA sequencing, which has enabled rapid pathogen identification to support the accurate diagnosis of infectious diseases at their earliest stages. In patients with pulmonary infections whose pathogens were not detected using traditional pathogen detection methods, a sequence-based assay developed by Huang et al. using this technology, that allowed in 94.49% samples successful detection of human pathogens. Moreover, their results showed that sequence-based tests were more accurate and sensitive than assays using standard pathogen detection [24]. In the mixed pulmonary infections detection, Wang et al. also identified sequence-based detection methods to be more sensitive than traditional methods [25]. In present study, we found that nanopore sequencing of PCR products enabled us to effectively diagnose suspected PTB cases, while also providing superior sensitivity (83.33%) as compared to sensitivities obtained with MGIT culture (36.11%) and Xpert MTB/RIF (40.28%). Furthermore, in the diagnosing of suspected PTB cases, in comparison with MGIT culture and Xpert MTB/RIF analysis, the nanopore sequencing had better performance in the diagnosis, as reflected by Youden’s index and area under the curve (AUC) values. In summarizing, above results indicate that nanopore sequencing holds promise to be a valuable additional assay to optimize the diagnostic detection of PTB cases, especially for the detection of TB and NTM organisms that cannot be distinguished the MGIT culture assay and thus produced positive MTB culture results for both mycobacterial types, with 3 of 16 NTM-containing control samples testing positive for MTB. For the 3 cases, PNB and CT were used to identify the possibility of co-infection and the effectiveness of treatment options. The PNB identification method is an effective microbiological tool that utilizes the fundamental differences between TB and NTM in metabolizing PNB. Through this method, these two types of bacteria can be effectively distinguished. On PNB selective medium, the growth of TB is inhibited, while NTM is able to grow, thus achieving separation and identification of the two. This high-number of false positive outcomes corroborated the low specificity of the MGIT culture test and indicated that when using this test for TB diagnosis in NTM epidemic areas, the results it provides should be interpreted thoroughly. Nevertheless, the specificity of MGIT culture assay was adapted to differentiate between mycobacterial and non-mycobacterial organisms.

With regard to assay specificity, Xpert only specifically targets the MTB complex-associated rpoB sequence, which exists in only one copy within the MTB genome. In contrast, through targeting the IS6110 insertion sequence (which is not present in NTM genomes) and thus nanopore sequencing analysis has specific for the M. tuberculosis complex. In addition, the IS6110 sequence is present at 10 to 12 copies per genome for diverse MTB strains and thus nanopore sequencing based on detection of this sequence can provide superior MTB-detection sensitivity when used to detect diverse MTB isolates that exhibit different tissue spread patterns and pathogenicity. In order to confirm the reliability of the nanopore sequencing results, we also used hsp65 and gyrB genes as the basis for distinguishing the Mycobacterium tuberculosis complex and NTM.

In addition, the limitations of this study include: firstly, due to the smaller sample size for this study, so the results obtained may be biased and therefore future diagnostic potency of nanopore sequencing studies should be investigated using a larger sample size. Second, the focus of this study was the detection of BALF samples, so lavage samples collected containing pathogen numbers below the assay lower limit of detection could lead to false negative results. Therefore, nanopore sequencing should still be viewed as an auxiliary diagnostic tool to be apply to conjunction clinical characteristics, radiology imaging results and other laboratory testing findings. Third, false positive and false negative nanopore sequencing analysis results cannot be excluded which caused by (1) the depths of sequence were too low; (2) the biomass of microbial pathogen was low while the background noise of host genome was high; (3) the patient was on antibiotics before the test; and (4) the samples were contaminated with human flora or environmental microorganisms [25]. We checked the specificity of the primer and found that it performed well in the identification of mycobacteria. The samples were loaded with human flora and environmental microorganisms. This was mainly because the background microorganisms in the samples were too rich, which made the amount of Mycobacterium tuberculosis nucleic acid (when the PCR input was 2–20 ng) relatively low, which may lead to false negative results. We will also optimize our experimental process in the future.Fourth, although one patient’s PCR products contained an antimicrobial drug resistance gene sequence, we did not obtain the patient’s antibiotic treatment result and thus could not determine whether the sequencing results of patients are aligned with treatment response. Fifth, patients with certain control diseases, like rheumatoid arthritis and lymphomas, were absence in our study cohort, which may have biased the results. However, the shell vial culture and nanopore sequencing assays of biopsied tissue samples could overcome this issue. Sixth, this study found that one patient’s PCR products contained an antimicrobial drug resistance gene sequence, and the role of nanopore sequencing in the direct detection of rifampicin and fluoroquinolone resistance was not evaluated. In future, a few drug-resistant smear negative BAL samples should be included to further investigate the potential application of nanopore sequencing in drug susceptibility testing in further studies. Compared with MGIT culture and Xpert MTB/RIF assays, BALF’s nanopore sequencing provided superior MTB detection sensitivity and thus is suitable for testing of sputum-scarce suspected PTB cases, which be used as a complement to MTB testing. Nevertheless, our findings need to be validated by including a larger and more diverse patient population for further studies.

## Data Availability

The datasets used or analyzed during the current study are available from the corresponding author on reasonable request.

## References

[1] World Health Organization. Global Tuberculosis Report 2021. Geneva.

[2] Gliddon HD, Frampton D, Munsamy V, Heaney J, Pataillot-Meakin T, Nastouli E (2021). A Rapid Drug Resistance Genotyping Workflow for Mycobacterium tuberculosis, using targeted isothermal amplification and nanopore sequencing. Microbiol Spectr.

[3] George S, Xu Y, Rodger G, Morgan M, Sanderson ND, Hoosdally SJ (2020). DNA Thermo-Protection facilitates whole-genome sequencing of Mycobacteria Direct from clinical samples. J Clin Microbiol.

[4] Votintseva AA, Bradley P, Pankhurst L, Del Ojo Elias C, Loose M, Nilgiriwala K (2017). Same-Day Diagnostic and Surveillance Data for Tuberculosis via whole-genome sequencing of direct respiratory samples. J Clin Microbiol.

[5] Liu Z, Yang Y, Wang Q, Wang L, Nie W, Chu N (2023). Diagnostic value of a nanopore sequencing assay of bronchoalveolar lavage fluid in pulmonary tuberculosis. BMC Pulm Med.

[6] Jonathon TH, Bradley LD, Brent WB, Su YC, Megan S, Yost HJ (2014). Poly peak parser: method and software for identifification of unknown indels using sanger sequencing of polymerase chain reaction products. Dev Dyn.

[7] Wouter DC, Svenn D, Schultz DT, Cruts M, van Broeckhoven C (2018).

[8] Geneva Switzerland Who Global Tuberculosis Programme. Sequence correction provided by ONT Research. 2021, https://github.com/nanoporetech/medaka.

[9] Chen LX, Cai Y, Zhou GB, Shi XJ, Su JH, Chen GW (2014). Rapid Sanger sequencing of the 16S rRNA gene for identifification of some common pathogens. PLoS ONE.

[10] Tewari D, Cieply S, Livengood J (2011). Identifification of bacteria recovered from animals using the 16S ribosomal RNA gene with pyrosequencing and sanger sequencing. J Vet Diagn Invest.

[11] Jarvie T (2005). Next generation sequencing technologies. Drug Discov Today Technol.

[12] Voelkerding KV, Dames SA, Durtschi JD (2009). Next-generation sequencing: from basic research to diagnostics. Clin Chem.

[13] Simner PJ, Miller S, Carroll KC (2018). Understanding the promises and hurdles of metagenomic next-generation sequencing as a diagnostic tool for infectious diseases. Clin Infect Dis.

[14] Wilson MR, Naccache SN, Samayoa E, Biagtan M, Bashir M, Yu G (2014). Actionable diagnosis of neuroleptospirosis by next-generation sequencing. N Engl J Med.

[15] Satta G, Lipman M, Smith GP, Arnold C, Kon OM, TD McHugh (2018). Mycobacterium tuberculosis and whole-genome sequencing: how close are we to unleashing its full potential. Clin Microbiol Infect.

[16] van Ingen J, Kohl TA, Kranzer K, Hasse B, Keller PM, Katarzyna SA (2017). Global outbreak of severe Mycobacterium chimaera disease after cardiac surgery: a molecular epidemiological study. Lancet Infect Dis.

[17] Wang L, Liu D, Yung L, Rodriguez GD, Prasad N, Segal-Maurer S (2021). Coinfection with 4 species of mycobacteria identifified by using next-generation sequencing. Emerg Infect Dis.

[18] Zhu H, Zhu M, Lei JH, Xiao YL, Zhao LM (2021). Metagenomic nextgeneration sequencing can clinch diagnosis of non-tuberculous mycobacterial infections: a case report. Front Med (Lausanne).

[19] Hendrix J, Epperson LE, Durbin D, Honda JR, Strong M (2021). Intraspecies plasmid and genomic variation of Mycobacterium kubicae revealed by the complete genome sequences of two clinical isolates. Microb Genom.

[20] Stefani MMA, Avanzi C, Bührer-Sékula S, Benjak A, Loiseau C, Singh P (2017). Whole genome sequencing distinguishes between relapse and reinfection in recurrent leprosy cases. PLoS Negl Trop Dis.

[21] Laver T, Harrison J, O’Neill PA, Moore K, Farbos A, Paszkiewicz K (2015). Assessing the performance of the oxford nanopore technologies MinION. Biomol Detect Quantif.

[22] Alvarez JR, Skachkov D, Massey SE, Kalitsov A, Velev JP (2015). DNA/RNA transverse current sequencing: intrinsic structural noise from neighboring bases. Front Genet.

[23] Rang FJ, Kloosterman WP, de Ridder J (2018). From squiggle to basepair: computational approaches for improving nanopore sequencing read accuracy. Genome Biol.

[24] Serpa PH, Deng X, Abdelghany M, Crawford E, Malcolm K, Caldera S (2022). Metagenomic prediction of antimicrobial resistance in critically ill patients with lower respiratory tract infections. Genome Med.

[25] Wang D, Wang W, Ding Y, Tang M, Zhang L, Chen J (2022). Metagenomic next-generation sequencing successfully detects PulmonaryInfectious pathogens in Children with Hematologic Malignancy. Front Cell Infect Microbiol.

